# (*E*)-6-Chloro-2-(furan-2-yl­methyl­idene)-2,3,4,9-tetra­hydro-1*H*-carbazol-1-one

**DOI:** 10.1107/S1600536810046714

**Published:** 2010-11-17

**Authors:** R. Archana, E. Yamuna, K. J. Rajendra Prasad, A. Thiruvalluvar, R. J. Butcher

**Affiliations:** a PG Research Department of Physics, Rajah Serfoji Government College (Autonomous), Thanjavur 613 005, Tamilnadu, India; b Department of Chemistry, Bharathiar University, Coimbatore 641 046, Tamilnadu, India; c Department of Chemistry, Howard University, 525 College Street NW, Washington, DC 20059, USA

## Abstract

In the title compound, C_17_H_12_ClNO_2_, the carbazole unit is nearly planar [maximum deviation = 0.052 (1) Å]. The pyrrole ring makes dihedral angles of 1.92 (8) and 4.71 (11)° with the benzene and furan rings, respectively. Inter­molecular N—H⋯O hydrogen bonds form *R*_2_^2^(10) rings in the crystal structure.

## Related literature

For the pharmaceutical inter­est of heteroaryl annulated derivatives of carbazoles, see: Knölker & Reddy (2002 ▶, 2008 ▶). For the preparation of various hetero-annulated carbazoles, see: Sridharan *et al.* (2008 ▶); Danish & Rajendra Prasad (2004 ▶, 2005 ▶). For hydrogen-bond motifs, see: Bernstein *et al.* (1995 ▶).
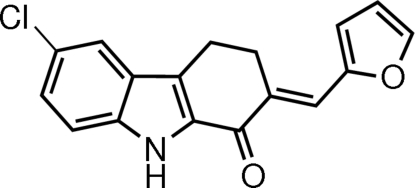


## Experimental

### 

#### Crystal data

C_17_H_12_ClNO_2_
*M* *_r_* = 297.73
Monoclinic, 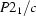

*a* = 15.0985 (2) Å
*b* = 6.1553 (1) Å
*c* = 15.3887 (2) Å
β = 104.319 (1)°
*V* = 1385.73 (3) Å^3^
*Z* = 4
Cu *K*α radiation
μ = 2.47 mm^−1^
*T* = 295 K
0.48 × 0.34 × 0.12 mm

#### Data collection

Oxford Diffraction Xcalibur Ruby Gemini diffractometer
Absorption correction: multi-scan (*CrysAlis PRO*; Oxford Diffraction, 2010 ▶) *T*_min_ = 0.389, *T*_max_ = 1.000
8660 measured reflections
2834 independent reflections
2676 reflections with *I* > 2σ(*I*)
*R*_int_ = 0.026

#### Refinement

*R*[*F* ^2^ > 2σ(*F* ^2^)] = 0.047
*wR*(*F* ^2^) = 0.139
*S* = 1.10
2834 reflections
194 parameters
H atoms treated by a mixture of independent and constrained refinement
Δρ_max_ = 0.28 e Å^−3^
Δρ_min_ = −0.28 e Å^−3^

### 

Data collection: *CrysAlis PRO* (Oxford Diffraction, 2010 ▶); cell refinement: *CrysAlis PRO*; data reduction: *CrysAlis PRO*; program(s) used to solve structure: *SHELXS97* (Sheldrick, 2008 ▶); program(s) used to refine structure: *SHELXL97* (Sheldrick, 2008 ▶); molecular graphics: *ORTEP-3* (Farrugia, 1997 ▶) and *PLATON* (Spek, 2009 ▶); software used to prepare material for publication: *PLATON*.

## Supplementary Material

Crystal structure: contains datablocks global, I. DOI: 10.1107/S1600536810046714/bq2253sup1.cif

Structure factors: contains datablocks I. DOI: 10.1107/S1600536810046714/bq2253Isup2.hkl

Additional supplementary materials:  crystallographic information; 3D view; checkCIF report

## Figures and Tables

**Table 1:** Hydrogen-bond geometry (Å, °)

*D*—H⋯*A*	*D*—H	H⋯*A*	*D*⋯*A*	*D*—H⋯*A*
N9—H9⋯O1^i^	0.88 (2)	1.94 (2)	2.7935 (17)	164 (2)

Symmetry code: (i) 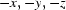.

**Fig. 1.**
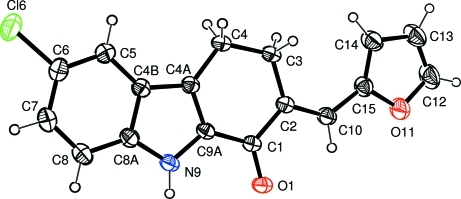
The molecular structure of the title compound, showing the atom-numbering scheme and displacement ellipsoids drawn at the 30% probability level.

**Fig. 2.**
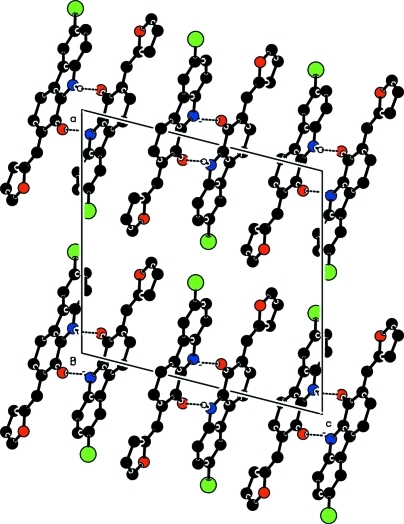
The molecular packing of the title compound, viewed down the b axis. Dashed lines indicate hydrogen bonds. H atoms not involved in hydrogen bonding have been omitted.

## supplementary crystallographic information

### Comment

Aryl and heteroarylcarbazoles are important classes of biologically active compounds that include notable alkaloids of pharmaceutical interest (Knölker & Reddy (2002, 2008)) and heteroaryl annulated derivatives of carbazole. From our laboratory, we have reported the synthesis of 2-benzylidene-2,3,4,9-tetrahydrocarbazoles from the precursors of the 2,3,4,9-tetrahydro-1*H*-carbazol-1-one type and these synthons were utilized to prepare many heteroannulated carbazoles (Sridharan *et al.*, (2008); Danish & Rajendra Prasad (2004, 2005)).

In the title molecule (Fig. 1), C_17_H_12_ClNO_2_, the carbazole unit is nearly planar [maximum deviation = 0.052 (1) Å for C1]. The pyrrole ring makes dihedral angles of 1.92 (8)° and 4.71 (11)° with the benzene and the furan rings, respectively. Intermolecular N9—H9···O1 hydrogen bonds form a *R*_2_^2^(10) (Bernstein *et al.*, 1995) ring in the crystal structure (Table 1, Fig. 2).

### Experimental

An equimolar mixture of 6-chloro-2,3,4,9-tetrahydro-1*H*-carbazol-1-one (1.095 g, 0.005 mol) and furan-2-carbaldehyde (0.41 ml, 0.005 mol) was treated with 25 ml of a 5% ethanolic potassium hydroxide solution and stirred for 6 h at room temperature. The product precipitated as a yellow crystalline mass, was filtered off and washed with 50% ethanol. A further crop of condensation product was obtained on neutralization with acetic acid and dilution with water. The product was recrystallized from methanol to yield 90% (1.336 g) of the title compound. The pure compound was recrystallized from EtOAc.

### Refinement

The H atom bonded to N9 was located in a difference Fourier map and refined freely. Other H atoms were positioned geometrically and allowed to ride on their parent atoms, with C—H = 0.93–0.97 Å and *U*_iso_(H) = 1.2*U*_eq_(parent atom).

### Crystal data

**Table:**

C_17_H_12_ClNO_2_	*F*(000) = 616
*M**_r_* = 297.73	*D*_x_ = 1.427 Mg m^−^^3^
Monoclinic, *P*2_1_/*c*	Melting point: 501 K
Hall symbol: -P 2ybc	Cu *K*α radiation, λ = 1.54184 Å
*a* = 15.0985 (2) Å	Cell parameters from 6923 reflections
*b* = 6.1553 (1) Å	θ = 4.7–75.4°
*c* = 15.3887 (2) Å	µ = 2.47 mm^−^^1^
β = 104.319 (1)°	*T* = 295 K
*V* = 1385.73 (3) Å^3^	Prism, pale-yellow
*Z* = 4	0.48 × 0.34 × 0.12 mm

### Data collection

**Table:**

Oxford Diffraction Xcalibur Ruby Gemini diffractometer	2834 independent reflections
Radiation source: Enhance (Cu) X-ray Source	2676 reflections with *I* > 2σ(*I*)
graphite	*R*_int_ = 0.026
Detector resolution: 10.5081 pixels mm^-1^	θ_max_ = 75.6°, θ_min_ = 5.9°
ω scans	*h* = −18→18
Absorption correction: multi-scan (*CrysAlis PRO*; Oxford Diffraction, 2010)	*k* = −5→7
*T*_min_ = 0.389, *T*_max_ = 1.000	*l* = −19→18
8660 measured reflections	

### Refinement

**Table:**

Refinement on *F*^2^	Primary atom site location: structure-invariant direct methods
Least-squares matrix: full	Secondary atom site location: difference Fourier map
*R*[*F*^2^ > 2σ(*F*^2^)] = 0.047	Hydrogen site location: inferred from neighbouring sites
*wR*(*F*^2^) = 0.139	H atoms treated by a mixture of independent and constrained refinement
*S* = 1.10	*w* = 1/[σ^2^(*F*_o_^2^) + (0.0845*P*)^2^ + 0.264*P*] where *P* = (*F*_o_^2^ + 2*F*_c_^2^)/3
2834 reflections	(Δ/σ)_max_ = 0.001
194 parameters	Δρ_max_ = 0.28 e Å^−^^3^
0 restraints	Δρ_min_ = −0.28 e Å^−^^3^

### Special details

**Table:**

Geometry. Bond distances, angles *etc*. have been calculated using the rounded fractional coordinates. All su's are estimated from the variances of the (full) variance-covariance matrix. The cell e.s.d.'s are taken into account in the estimation of distances, angles and torsion angles
Refinement. Refinement of *F*^2^ against ALL reflections. The weighted *R*-factor *wR* and goodness of fit *S* are based on *F*^2^, conventional *R*-factors *R* are based on *F*, with *F* set to zero for negative *F*^2^. The threshold expression of *F*^2^ > 2σ(*F*^2^) is used only for calculating *R*-factors(gt) *etc*. and is not relevant to the choice of reflections for refinement. *R*-factors based on *F*^2^ are statistically about twice as large as those based on *F*, and *R*- factors based on ALL data will be even larger.

### Fractional atomic coordinates and isotropic or equivalent isotropic displacement parameters (Å^2^)

**Table:**

	*x*	*y*	*z*	*U*_iso_*/*U*_eq_	
Cl6	−0.40900 (3)	0.76934 (9)	0.02641 (5)	0.0804 (2)	
O1	0.10076 (8)	0.13185 (18)	0.07979 (8)	0.0510 (3)	
O11	0.38257 (9)	0.4505 (3)	0.24198 (10)	0.0751 (5)	
N9	−0.09205 (9)	0.2203 (2)	0.03653 (8)	0.0441 (4)	
C1	0.07175 (10)	0.2982 (2)	0.10858 (9)	0.0376 (4)	
C2	0.13341 (10)	0.4564 (2)	0.16694 (8)	0.0383 (4)	
C3	0.09534 (11)	0.6580 (2)	0.20028 (10)	0.0457 (4)	
C4	−0.00564 (12)	0.7036 (3)	0.17066 (13)	0.0560 (5)	
C4A	−0.06155 (10)	0.5338 (2)	0.11436 (8)	0.0373 (4)	
C4B	−0.15750 (10)	0.5216 (2)	0.07663 (9)	0.0394 (4)	
C5	−0.23081 (11)	0.6625 (3)	0.07672 (10)	0.0476 (5)	
C6	−0.31602 (11)	0.5984 (3)	0.02964 (12)	0.0535 (5)	
C7	−0.33227 (11)	0.3997 (3)	−0.01636 (12)	0.0559 (5)	
C8	−0.26184 (12)	0.2604 (3)	−0.01737 (11)	0.0524 (5)	
C8A	−0.17369 (10)	0.3230 (3)	0.02874 (9)	0.0420 (4)	
C9A	−0.02410 (10)	0.3493 (2)	0.08760 (8)	0.0374 (4)	
C10	0.22300 (11)	0.4106 (3)	0.18622 (10)	0.0463 (4)	
C12	0.44470 (14)	0.5930 (5)	0.28938 (17)	0.0812 (8)	
C13	0.40378 (15)	0.7606 (4)	0.31507 (16)	0.0731 (7)	
C14	0.30864 (14)	0.7275 (3)	0.28188 (14)	0.0621 (6)	
C15	0.29763 (11)	0.5361 (3)	0.23740 (10)	0.0496 (5)	
H3A	0.12689	0.78195	0.18310	0.0548*	
H3B	0.11173	0.65244	0.26529	0.0548*	
H4A	−0.01436	0.83906	0.13742	0.0672*	
H4B	−0.02855	0.72442	0.22366	0.0672*	
H5	−0.22178	0.79380	0.10752	0.0571*	
H7	−0.39159	0.36229	−0.04650	0.0671*	
H8	−0.27226	0.12849	−0.04769	0.0629*	
H9	−0.0853 (14)	0.102 (4)	0.0073 (14)	0.060 (5)*	
H10	0.23867	0.28020	0.16336	0.0556*	
H12	0.50773	0.57447	0.30194	0.0974*	
H13	0.43190	0.87846	0.34865	0.0877*	
H14	0.26237	0.82010	0.28928	0.0746*	

### Atomic displacement parameters (Å^2^)

**Table:**

	*U*^11^	*U*^22^	*U*^33^	*U*^12^	*U*^13^	*U*^23^
Cl6	0.0463 (3)	0.0751 (4)	0.1142 (5)	0.0130 (2)	0.0090 (3)	0.0023 (3)
O1	0.0491 (6)	0.0413 (6)	0.0619 (6)	0.0001 (4)	0.0123 (5)	−0.0157 (5)
O11	0.0436 (7)	0.0848 (10)	0.0917 (10)	0.0015 (6)	0.0071 (6)	−0.0262 (8)
N9	0.0461 (7)	0.0415 (6)	0.0439 (6)	−0.0039 (5)	0.0094 (5)	−0.0113 (5)
C1	0.0457 (8)	0.0334 (6)	0.0347 (6)	−0.0031 (5)	0.0121 (5)	−0.0023 (5)
C2	0.0441 (7)	0.0355 (7)	0.0357 (6)	−0.0033 (5)	0.0106 (5)	−0.0015 (5)
C3	0.0483 (8)	0.0383 (7)	0.0485 (8)	−0.0044 (6)	0.0084 (6)	−0.0116 (6)
C4	0.0495 (9)	0.0438 (8)	0.0696 (10)	0.0021 (7)	0.0049 (8)	−0.0228 (7)
C4A	0.0427 (7)	0.0362 (7)	0.0326 (6)	−0.0011 (5)	0.0088 (5)	0.0002 (5)
C4B	0.0432 (7)	0.0401 (7)	0.0348 (6)	−0.0018 (5)	0.0094 (5)	0.0009 (5)
C5	0.0476 (8)	0.0445 (8)	0.0502 (8)	0.0019 (6)	0.0113 (6)	−0.0001 (6)
C6	0.0438 (8)	0.0574 (9)	0.0578 (9)	0.0045 (7)	0.0097 (7)	0.0074 (7)
C7	0.0424 (8)	0.0650 (10)	0.0563 (9)	−0.0074 (7)	0.0044 (7)	−0.0001 (8)
C8	0.0491 (9)	0.0553 (9)	0.0501 (8)	−0.0104 (7)	0.0070 (7)	−0.0082 (7)
C8A	0.0445 (8)	0.0439 (7)	0.0372 (6)	−0.0037 (6)	0.0093 (5)	−0.0017 (5)
C9A	0.0449 (7)	0.0358 (7)	0.0316 (6)	−0.0045 (5)	0.0094 (5)	−0.0030 (5)
C10	0.0467 (8)	0.0452 (8)	0.0473 (7)	−0.0023 (6)	0.0120 (6)	−0.0080 (6)
C12	0.0431 (9)	0.1089 (19)	0.0868 (14)	−0.0143 (11)	0.0071 (9)	−0.0239 (14)
C13	0.0570 (11)	0.0854 (14)	0.0749 (12)	−0.0234 (10)	0.0127 (9)	−0.0238 (11)
C14	0.0527 (10)	0.0670 (11)	0.0673 (10)	−0.0102 (8)	0.0160 (8)	−0.0205 (9)
C15	0.0418 (8)	0.0585 (9)	0.0484 (8)	−0.0023 (7)	0.0112 (6)	−0.0053 (7)

### Geometric parameters (Å, °)

**Table:**

Cl6—C6	1.7452 (18)	C6—C7	1.404 (3)
O1—C1	1.2380 (17)	C7—C8	1.369 (3)
O11—C12	1.358 (3)	C8—C8A	1.399 (2)
O11—C15	1.372 (2)	C10—C15	1.431 (2)
N9—C8A	1.364 (2)	C12—C13	1.313 (4)
N9—C9A	1.3791 (19)	C13—C14	1.415 (3)
N9—H9	0.88 (2)	C14—C15	1.352 (3)
C1—C9A	1.438 (2)	C3—H3A	0.9700
C1—C2	1.4859 (19)	C3—H3B	0.9700
C2—C3	1.5098 (19)	C4—H4A	0.9700
C2—C10	1.341 (2)	C4—H4B	0.9700
C3—C4	1.506 (3)	C5—H5	0.9300
C4—C4A	1.481 (2)	C7—H7	0.9300
C4A—C4B	1.423 (2)	C8—H8	0.9300
C4A—C9A	1.3763 (18)	C10—H10	0.9300
C4B—C8A	1.417 (2)	C12—H12	0.9300
C4B—C5	1.407 (2)	C13—H13	0.9300
C5—C6	1.369 (2)	C14—H14	0.9300
			
Cl6···C12^i^	3.613 (3)	C3···H14	2.7400
O1···N9	2.8733 (19)	C5···H14^v^	3.0700
O1···N9^ii^	2.7935 (17)	C9A···H4B^v^	2.9200
O1···H3A^iii^	2.6500	C14···H3A	2.8100
O1···H9	2.76 (2)	C14···H3B	2.9600
O1···H10	2.3400	C15···H3B	3.0300
O1···H4A^iii^	2.8000	C15···H3A	2.9300
O1···H9^ii^	1.94 (2)	H3A···O1^vi^	2.6500
N9···O1	2.8733 (19)	H3A···C14	2.8100
N9···O1^ii^	2.7935 (17)	H3A···C15	2.9300
N9···H4A^iii^	2.9000	H3A···H14	2.2900
C1···C4A^iv^	3.5499 (18)	H3B···C14	2.9600
C1···C4B^iv^	3.585 (2)	H3B···C15	3.0300
C2···C8A^iv^	3.4910 (19)	H3B···H14	2.4400
C3···C14	3.183 (3)	H4A···O1^vi^	2.8000
C4A···C1^iv^	3.5499 (18)	H4A···N9^vi^	2.9000
C4B···C1^iv^	3.585 (2)	H4A···H9^vi^	2.5900
C7···C15^iv^	3.589 (2)	H4B···C1^i^	2.8500
C8···C10^iv^	3.457 (2)	H4B···C2^i^	2.9500
C8···C15^iv^	3.524 (2)	H4B···C9A^i^	2.9200
C8A···C2^iv^	3.4910 (19)	H9···O1	2.76 (2)
C9A···C9A^iv^	3.4944 (18)	H9···H4A^iii^	2.5900
C10···C8^iv^	3.457 (2)	H9···O1^ii^	1.94 (2)
C12···Cl6^v^	3.613 (3)	H9···C1^ii^	3.08 (2)
C14···C3	3.183 (3)	H10···O1	2.3400
C15···C8^iv^	3.524 (2)	H14···C3	2.7400
C15···C7^iv^	3.589 (2)	H14···H3A	2.2900
C1···H4B^v^	2.8500	H14···H3B	2.4400
C1···H9^ii^	3.08 (2)	H14···C5^i^	3.0700
C2···H4B^v^	2.9500		
			
C12—O11—C15	107.00 (18)	O11—C12—C13	110.8 (2)
C8A—N9—C9A	108.18 (12)	C12—C13—C14	106.8 (2)
C9A—N9—H9	127.4 (14)	C13—C14—C15	107.15 (18)
C8A—N9—H9	123.9 (14)	C10—C15—C14	136.93 (18)
O1—C1—C9A	121.75 (13)	O11—C15—C10	114.82 (16)
O1—C1—C2	122.28 (14)	O11—C15—C14	108.20 (16)
C2—C1—C9A	115.97 (11)	C2—C3—H3A	107.00
C3—C2—C10	123.06 (13)	C2—C3—H3B	107.00
C1—C2—C3	120.65 (13)	C4—C3—H3A	107.00
C1—C2—C10	116.28 (13)	C4—C3—H3B	107.00
C2—C3—C4	119.50 (13)	H3A—C3—H3B	107.00
C3—C4—C4A	115.69 (14)	C3—C4—H4A	108.00
C4B—C4A—C9A	106.65 (11)	C3—C4—H4B	108.00
C4—C4A—C4B	130.64 (13)	C4A—C4—H4A	108.00
C4—C4A—C9A	122.71 (14)	C4A—C4—H4B	108.00
C5—C4B—C8A	119.86 (14)	H4A—C4—H4B	107.00
C4A—C4B—C5	133.55 (13)	C4B—C5—H5	121.00
C4A—C4B—C8A	106.56 (12)	C6—C5—H5	121.00
C4B—C5—C6	117.29 (16)	C6—C7—H7	120.00
C5—C6—C7	122.82 (16)	C8—C7—H7	120.00
Cl6—C6—C5	119.03 (14)	C7—C8—H8	121.00
Cl6—C6—C7	118.15 (13)	C8A—C8—H8	121.00
C6—C7—C8	120.79 (16)	C2—C10—H10	116.00
C7—C8—C8A	117.78 (16)	C15—C10—H10	116.00
N9—C8A—C8	130.00 (16)	O11—C12—H12	125.00
C4B—C8A—C8	121.44 (15)	C13—C12—H12	125.00
N9—C8A—C4B	108.55 (13)	C12—C13—H13	127.00
C1—C9A—C4A	125.36 (12)	C14—C13—H13	127.00
N9—C9A—C1	124.59 (12)	C13—C14—H14	126.00
N9—C9A—C4A	110.05 (13)	C15—C14—H14	126.00
C2—C10—C15	128.47 (16)		
			
C15—O11—C12—C13	0.5 (3)	C9A—C4A—C4B—C8A	0.88 (15)
C12—O11—C15—C10	177.58 (17)	C4—C4A—C9A—N9	179.63 (13)
C12—O11—C15—C14	−0.2 (2)	C4—C4A—C9A—C1	−1.0 (2)
C9A—N9—C8A—C4B	−0.48 (16)	C4B—C4A—C9A—N9	−1.21 (15)
C9A—N9—C8A—C8	178.81 (16)	C4B—C4A—C9A—C1	178.14 (12)
C8A—N9—C9A—C1	−178.29 (13)	C4A—C4B—C5—C6	177.85 (15)
C8A—N9—C9A—C4A	1.07 (15)	C8A—C4B—C5—C6	0.2 (2)
O1—C1—C2—C3	179.69 (13)	C4A—C4B—C8A—N9	−0.25 (16)
O1—C1—C2—C10	0.7 (2)	C4A—C4B—C8A—C8	−179.62 (14)
C9A—C1—C2—C3	−0.52 (18)	C5—C4B—C8A—N9	177.96 (13)
C9A—C1—C2—C10	−179.50 (13)	C5—C4B—C8A—C8	−1.4 (2)
O1—C1—C9A—N9	1.5 (2)	C4B—C5—C6—Cl6	−179.12 (12)
O1—C1—C9A—C4A	−177.73 (13)	C4B—C5—C6—C7	1.1 (3)
C2—C1—C9A—N9	−178.27 (12)	Cl6—C6—C7—C8	179.00 (14)
C2—C1—C9A—C4A	2.48 (19)	C5—C6—C7—C8	−1.2 (3)
C1—C2—C3—C4	−2.7 (2)	C6—C7—C8—C8A	−0.1 (3)
C10—C2—C3—C4	176.20 (15)	C7—C8—C8A—N9	−177.91 (16)
C1—C2—C10—C15	177.45 (15)	C7—C8—C8A—C4B	1.3 (2)
C3—C2—C10—C15	−1.5 (2)	C2—C10—C15—O11	−177.11 (16)
C2—C3—C4—C4A	4.0 (2)	C2—C10—C15—C14	−0.1 (3)
C3—C4—C4A—C4B	178.72 (14)	O11—C12—C13—C14	−0.5 (3)
C3—C4—C4A—C9A	−2.3 (2)	C12—C13—C14—C15	0.4 (3)
C4—C4A—C4B—C5	2.1 (3)	C13—C14—C15—O11	−0.1 (2)
C4—C4A—C4B—C8A	179.97 (15)	C13—C14—C15—C10	−177.2 (2)
C9A—C4A—C4B—C5	−176.98 (15)		

Symmetry codes: (i) −*x*, *y*+1/2, −*z*+1/2; (ii) −*x*, −*y*, −*z*; (iii) *x*, *y*−1, *z*; (iv) −*x*, −*y*+1, −*z*; (v) −*x*, *y*−1/2, −*z*+1/2; (vi) *x*, *y*+1, *z*.

### Hydrogen-bond geometry (Å, °)

**Table:**

*D*—H···*A*	*D*—H	H···*A*	*D*···*A*	*D*—H···*A*
N9—H9···O1^ii^	0.88 (2)	1.94 (2)	2.7935 (17)	164 (2)

Symmetry codes: (ii) −*x*, −*y*, −*z*.
